# DNA Damage and Reactive Nitrogen Species are Barriers to *Vibrio cholerae* Colonization of the Infant Mouse Intestine

**DOI:** 10.1371/journal.ppat.1001295

**Published:** 2011-02-17

**Authors:** Bryan W. Davies, Ryan W. Bogard, Nicole M. Dupes, Tyler A. I. Gerstenfeld, Lyle A. Simmons, John J. Mekalanos

**Affiliations:** 1 Department of Microbiology and Molecular Genetics, Harvard Medical School, Boston, Massachusetts, United States of America; 2 Department of Molecular, Cellular, and Developmental Biology, University of Michigan, Ann Arbor, Michigan, United States of America; Yale University School of Medicine, United States of America

## Abstract

Ingested *Vibrio cholerae* pass through the stomach and colonize the small intestines of its host. Here, we show that *V. cholerae* requires at least two types of DNA repair systems to efficiently compete for colonization of the infant mouse intestine. These results show that *V. cholerae* experiences increased DNA damage in the murine gastrointestinal tract. Agreeing with this, we show that passage through the murine gut increases the mutation frequency of *V. cholerae* compared to liquid culture passage. Our genetic analysis identifies known and novel defense enzymes required for detoxifying reactive nitrogen species (but not reactive oxygen species) that are also required for *V. cholerae* to efficiently colonize the infant mouse intestine, pointing to reactive nitrogen species as the potential cause of DNA damage. We demonstrate that potential reactive nitrogen species deleterious for *V. cholerae* are not generated by host inducible nitric oxide synthase (iNOS) activity and instead may be derived from acidified nitrite in the stomach. Agreeing with this hypothesis, we show that strains deficient in DNA repair or reactive nitrogen species defense that are defective in intestinal colonization have decreased growth or increased mutation frequency in acidified nitrite containing media. Moreover, we demonstrate that neutralizing stomach acid rescues the colonization defect of the DNA repair and reactive nitrogen species defense defective mutants suggesting a common defense pathway for these mutants.

## Author Summary

Studies on intracellular bacterial pathogens have shown the need for maintaining genomic fidelity to promote colonization. Loss of DNA repair functions often leads to attenuation and rapid clearing of the invading pathogen. However, for some pathogens, an increased mutation rate has been shown to be beneficial for promoting host colonization, presumably by allowing the pathogen to rapidly adapt to adverse host conditions. We asked if the non-invasive pathogen *V. cholerae* experienced increased DNA damage during infection and if so, how the increased damage influenced host colonization and from where the source of the damage was derived. Our results demonstrate that *V. cholerae* experiences increased DNA damage during infection in the infant mouse model and that loss of ability to repair this damage results in attenuation of virulence. We specifically show that *V. cholerae* requires both base excision repair and mismatch repair for efficient intestinal colonization. Furthermore, we present evidence that the source of the DNA damage is derived from reactive nitrogen species (RNS) formed by acidified nitrite in the mouse gut and in doing so we identify a new RNS defense protein found in *V. cholerae* and several other pathogenic bacteria.

## Introduction

Maintaining genomic integrity during infection is important for several bacterial pathogens to colonize their hosts. DNA repair defects in *Listeria monocytogenes*, *Salmonella typhimurium*, *Helicobacter pylori* and others leads to decreased or even a complete attenuation of virulence [1]–[6]. While there are several types of DNA repair in bacteria [7], many of the studies showing a requirement for DNA repair in pathogenesis focus on three pathways: the SOS response, base excision repair, and mismatch repair [1]–[10].

The SOS response is a well studied and conserved stress response in bacteria that is elicited following DNA damage and replication fork arrest [for review [11]]. The SOS response is controlled by positive and negative regulators. RecA positively regulates the SOS response by binding to single-stranded DNA fragments generated by attempted replication past DNA lesions. The RecA/ssDNA nucleoprotein filament induces the auto-cleavage of the negative regulator LexA, a transcriptional repressor. Cleavage of LexA allows for expression of 57 genes in the *E. coli* SOS regulon including translesion DNA polymerases that are able to replicate DNA past noncoding base lesion, and proteins involved in the inhibition of cell division [for review [11]].

Base excision repair (BER) is the most common form of repair for single base damage [for review [7]]. In BER, a DNA N-glycosylase first excises the damaged base from the deoxyribose moiety in the DNA strand creating an abasic site. Class II apurinic/apyrimidinic (AP) endonuclease then hydrolyzes the phosphodiester bond immediately 5′ to the abasic site [for review (Kornberg and Baker 1992, Friedberg 2005)]. Subsequent actions process this site to prime and repair the abasic site ultimately by DNA synthesis with DNA polymerase I and ligation by DNA ligase.

Normal replication can also introduce errors in the form of mismatched DNA base-pairs. These mismatches can lead to permanent mutations after a subsequent round of DNA replication. Mismatch repair (MMR) specifically identifies and corrects these base pairing errors increasing the fidelity of the replication pathway nearly ∼1000-fold [for review [7]].

While extensive work has shown the benefit of maintaining genomic integrity for an invading bacterium, there appear to be instances where lapses in genomic fidelity are beneficial for a pathogenic bacterium [12]–[14]. In order to colonize and thrive in a mammalian host, a bacterium must be able to adapt and respond to the conditions and stresses associated its new environment. Genomic mutations support this by allowing current gene products to gain or alter their functions. The utility of mutation(s) and a pathogen's ability to grow in the human environment has been a source of discussion for several years [13], [15]. Giraud *et al.* showed that a high mutation rate was initially beneficial for *Escherichia coli* to colonize the mouse gut, but this benefit became a liability once adaptation had been reached [12]. Oliver *et al.* demonstrated that *Pseudomonas aeruginosa* from chronically infected individuals often has an increased mutation frequency, suggesting an increased mutation rate can be beneficial to *P. aeruginosa* to allow rapid adaptation to the hostile host environment [14]. Thus depending on the pathogen, the mode and duration of the infection, defects in DNA repair may be detrimental or beneficial to the infecting bacterium.

Several studies have indicated that host produced reactive oxygen species (ROS) and reactive nitrogen species (RNS) cause DNA damage to the invading bacterium [4], [16], [17]. Not surprisingly bacteria have several defense mechanisms to detoxify ROS and RNS. Each enzyme detoxifies a specific type of ROS or RNS. For example catalases/peroxidases decompose H_2_O_2_, superoxide dismutases dismutate superoxide and ferrisiderophore reductase removes nitric oxide [18], [19]. As with certain DNA repair systems, loss of ROS and RNS defenses have been shown to attenuate bacterial pathogens [16], [20].

Studies supporting the importance of ROS/RNS defenses and DNA repair pathways in bacterial pathogenesis often focus on intracellular pathogens [1], [2], [4]. To survive, intracellular pathogens engulfed by phagocytic cells are either able to escape the phagosome or have mechanisms to survive within it. Within the phagosome, captured bacteria may be exposed to host production of ROS and RNS in a host defense response called the oxidative burst. It is hypothesized that the oxidative burst is responsible for the DNA damage experienced by engulfed bacteria [4], [16], [17].

*Vibrio cholerae* is the causative agent of the severe human diarrheal disease cholera. *V. cholerae* is a non-invasive pathogen that colonizes the small intestine of its host [21], [22]. As a non-invasive pathogen, *V. cholerae* is not expected to experience the same types of stresses as intracellular pathogens, such as an oxidative burst. However *V. cholerae* does pass through several hostile environments as the disease progresses. Immediately following ingestion, *V. cholerae* is exposed to the exceptionally antagonistic environment of the stomach where the pH of gastric acid can reach as low as 1 [23], [24]. Furthermore, nitrite from both food sources and the salivary nitrite cycle can enter the stomach creating acidified nitrite [25], [26], [27]. Acidified nitrite has potent antimicrobial effects on gut pathogens [28], [29], [30], [31]. These studies show that the viability of several pathogenic bacteria decreases rapidly under acidified nitrite conditions. Furthermore, nitrates, which can also be found in the stomach, have been shown to modify of gene expression reducing acid tolerance [32]. The antimicrobial effects of acidified nitrite are thought to be due to the generation of deleterious RNS [33]. However, with the exception of a few studies [34], [35], [36], the points of action of these RNS as well as the bacterial determinants required for protection against them have remained largely unexamined. After traversing the stomach *V. cholerae* faces several innate host defenses in the intestine including bile, lysozyme, small antimicrobial peptides and complement [37]. Thus, *V. cholerae* must overcome several barriers during infection that have the potential to cause DNA damage through a direct or indirect mechanism.

We report here that *V. cholerae* strain C6706 experiences increased DNA damage during passage through the murine gastrointestinal track. We demonstrate that increased genomic stress is a potential barrier to host colonization by *V. cholerae*. We found that two important DNA repair pathways are necessary for *V. cholerae* to efficiently colonize the infant mouse intestine. Furthermore, we show that defense against RNS is also necessary for *V. cholerae* to colonize the infant mouse. In doing so we identify a novel protein required for defense against RNS in pathogenic bacteria. *In vitro* we show that all our colonization defective DNA repair and RNS defense mutants share a common sensitivity to acidified nitrite and we further show that neutralizing stomach acid rescues intestinal colonization defect of these mutants.

## Results

### Mismatch repair and base excision repair pathways are required for *V. cholerae* colonization of the infant mouse intestine

To determine if *V. cholerae* requires defenses against DNA damage during colonization, we tested a series of transposon mutants that contained insertions in different steps in three important DNA repair pathways for their ability to colonize the infant mouse intestine in competition with the wild type strain. These pathways were nucleotide excision repair (NER), base excision repair (BER) and mismatch repair (MMR) (Table 1). While the SOS response is an important contributor to genomic integrity we did not test a requirement for SOS since Quiones *et al.* previously showed that SOS activation is not required for intestinal cholera toxin production or colonization [38]. We used *uvrA* as a representative gene required for NER since *uvrA* is obligatory for NER. We found no difference in the ability of the *uvrA*::*Tn* containing strain to colonize the infant mouse relative to the parental strain suggesting that NER is dispensable for *V. cholerae* pathogeneis (Table 1).

**Table 1:** Ability of *V. cholerae* mutants defective in DNA repair pathways to colonize the infant mouse intestine in competition with the parental strain (WT).

Gene	Function	Repair Pathway	aCompetitive Index: Tn Mutant/WT	aCompetitive Index: Deletion Mutant/WT
*uvrA*	Excinuclease ABC	Nucleotide Excision Repair	1.14±0.11	
*xth*	Exonuclease III	Base Excision Repair	1.07±0.05	
*nfo*	Endonuclease IV	Base Excision Repair	0.15±0.01^***^	0.23±0.01^***^
*xth/nfo*	Double mutant	Base Excision Repair	N/A	0.07±0.06^***^
*mutS*	Mismatch Recognition	Mismatch Repair	0.01±0.01^***^	0.16±0.07^***^
*nfo/mutS*	Double mutant	Base Excision and Mismatch Repair		0.08±0.02^***^

a The competitive index is the ratio of mutant to parental (WT) cfu in the small intestine post infection divided by the input ratio of mutant to parental (WT) cfu. The average and standard error of 5–7 mouse experiments is shown for each mutant. The *ΔmutS*, *Δnfo*, *xth*::Tn Δ*nfo*, *ΔmutS*, *Δnfo* mutant strains grew similarly to the wild type under free-living conditions (Figure S1A). The mean and standard error of 5–7 mouse experiments is shown for each mutant. Statistical significance of the competitive colonization defect of each mutant strain relative to the null hypothesis was determined as described in the materials and methods (*** p<0.001).

Apurinic/apyrimidininc (AP) endonucleases are critical in BER. BER has been most well studied in *E. coli*. *E. coli* encodes two class II AP endonucleases, Xth [endo II (endo VI)] and Nfo (endo IV). In *E. coli* Xth is responsible for ∼90% of the AP endonuclease activity in the cell [39], [40]. Few phenotypes have been attributed solely to Nfo activity but Nfo is known to contribute to BER [41]. *V. cholerae* carries close homologs of both Xth and Nfo (VC1860 and VC2360 respectively). Interestingly we found that the *xth*::Tn mutant was not defective in intestinal colonization however the *nfo*::Tn mutant showed a defect in colonization compared to the parental strains (Table 1). We created a clean deletion of *nfo* (Δ*nfo*) in *V. cholerae* and found this mutant also had a colonization defect. In *E. coli* deletion of *xth* and *nfo* leads to a more profound defect in DNA repair than either single mutant. Consistent with this observation, we found that an *xth*::Tn Δ*nfo* double mutant showed a ∼10-fold defect in colonization that appears slightly greater than the ∼5-fold defect in colonization of the Δ*nfo* mutant alone (Table 1), although this difference is not statistically significant for this number of replicates tested (p>0.05). Thus, these results suggest that BER is important for *V. cholerae* to colonize the infant mouse intestine when in competition. These results also show a critical function for Nfo in survival, which has not been apparent under laboratory conditions.

Loss of mismatch repair function has been shown to be either beneficial or detrimental depending on the pathogen studied [12], [13], [14], [15]. We found that a transposon mutant in *mutS*, which encodes the gene product that initially binds to a mismatch, resulted in a decrease in colonization efficiency (Table 1). We constructed a clean deletion of *mutS* (*ΔmutS*) to ensure the defect was not due to the transposon. We found that the clean deletion of *mutS* was also attenuated in its ability to colonize the intestine suggesting that mismatch repair or at least MutS is important for *V. cholerae* pathogenesis (Table 1). We also found that a second clean deletion of *mutS* showed a similar competitive index defect (CI = 0.18±0.03) suggesting that the colonization defect was not due to mutations in the first *mutS* clean deletion strain. We noted that the *mutS* transposon mutant was more defective than its clean deletion counterpart (Table 1). This difference may be due to a polar effect of the transposon or mutations acquired by the *mutS*::Tn strain during outgrowth of the original isolate. We also tested the colonization proficiency of a *Δnfo ΔmutS* double mutant and found that the colonization defect of this double mutant appears slightly greater than either the *Δnfo* or *ΔmutS* mutant alone (Table 1) although this difference is not statistically significant for this number of replicates tested (p>0.05).

The requirement of BER and MMR for *V. cholerae* to efficiently colonize the infant mouse intestine suggests that *V. cholerae* experiences DNA damage in the mouse, and that a reduced ability to repair such damage is detrimental for *V. cholerae* pathogenesis.

### *V. cholerae* base excision repair and mismatch repair mutants show classic DNA repair defects

**Figure 1:**
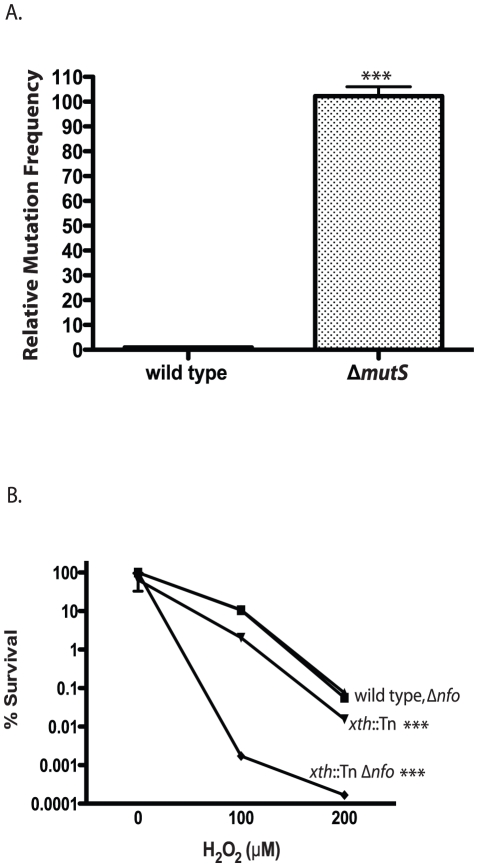
Phenotypes of *V. cholerae* DNA repair mutants. A. Mutation frequency. The number of Δ*mutS* rifampicin resistant colonies relative to wild type colonies is shown. The number of *V. cholerae* colonies was normalized to 1. The error bars reflect the SEM from at least 3 independent experiments (*** p<0.001). B. Hydrogen peroxide sensitivity. The sensitivity of wild type (▪), *xth*::Tn (▾), Δ*nfo* (▴) and *xth*::Tn Δ*nfo* (♦) strains to increasing concentrations of hydrogen peroxide are shown ± SEM from at least 3 independent experiments. The *xth*::Tn mutant is statistically different from the wild type and the Δ*nfo* mutant at 100µM and 200µM H_2_O_2_ (*** p<0.001). The *xth*::Tn Δ*nfo* strain is significantly different from the wild type, *xth*::Tn and Δ*nfo* mutant at 100µM and 200µM H_2_O_2_ (*** p<0.001).

*V. cholerae* genes encoding Xth, Nfo and MutS were identified based on sequence similarity with their well-studied *E. coli* homologs. To ensure the *V. cholerae* homologs possessed their predicted functions we tested our mutant strains for the well characterized phenotypes described in other bacterial systems. Loss of mismatch repair causes an increase in mutation rate often referred to as a mutator phenotype [42]. We found that our Δ*mutS* mutant had a significantly increased mutation frequency compared with the wild type control (Figure 1A). The wild type phenotype could be restored by expression of *mutS* from a plasmid but not by the plasmid itself (Figure S1B). This result indicates that MutS in *V. cholerae* shares the same activity as its other well studied bacterial homologs in the repair of DNA replication errors.

Loss of Xth activity in *E. coli* renders the strain sensitive to hydrogen peroxide (H_2_O_2_) [43]. We found that our *xth*::Tn strain was also sensitive to H_2_O_2_ (Figure 1B). Loss of *nfo* activity alone does not greatly sensitize *E. coli* to H_2_O_2_ but loss of *xth* and *nfo* creates a strain with increased sensitivity to H_2_O_2_ [43]. We found a similar effect in *V. cholerae* where the *xth*::Tn Δ*nfo* strain was much more sensitive to H_2_O_2_ then the *xth*::Tn mutant alone (Figure 1B). Furthermore, high level expression of *nfo* from a plasmid complemented the H_2_O_2_ sensitivity of the *xth*::Tn Δ*nfo* mutant (Figure S1C). These results suggest that *V. cholerae* Nfo acts like its *E. coli* homolog.

### Passage through the mouse increases the mutation frequency of *V. cholerae*

**Figure 2:**
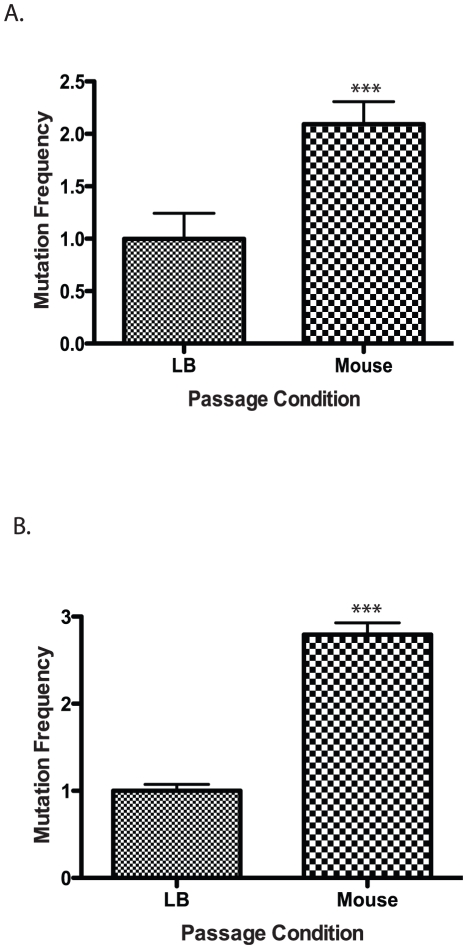
Mutation frequency of culture vs. mouse passaged wild type *V. cholerae*. Wild type cells were grown in LB or passaged through a mouse and plated on (A) rifampicin or (B) trimethopirin to determine the number of resistant colonies. The results show the average mutation frequency of *V. cholerae* from 5 mice relative to the average mutation frequency from 5 LB grown cultures. The average mutation frequency of the LB grown *V. cholerae* cultures was normalized to 1. The error bars reflect the SEM from at least 3 independent experiments (*** p<0.001).

The requirement of BER and mismatch repair (MMR) systems for *V. cholerae* to efficiently colonize the mouse intestine suggests that *V. cholerae* experiences increased DNA damage while in the mouse. To address this possibility we measured the mutation frequency of *V. cholerae* following passage though the mouse as compared to passage in liquid culture. We inoculated five mice and five liquid cultures with the same size inoculums of *V. cholerae*. The following day we purified bacteria from the mouse intestine (see Materials and Methods). We plated both *V. cholerae* passaged through the mouse and grown in liquid cultures followed by selection for resistance to two antibiotics we used as an indicator for measuring mutation frequency. The first was a gain of function mutation in *rpoB* conferring resistance to rifampicin; the second was a loss of function of *thyA* conferring resistance to trimethoprim. Mutations in *rpoB* and *thyA* are well characterized markers for increases in mutation frequency [44], [45], [46]. We found that following passage of *V. cholerae* through the mouse there was an ∼2 fold increase in rifampicin resistance and ∼2.5 fold increase in trimethoprim resistance compared to the liquid culture grown strains (Figure 2A, B). We sequenced 19 trimethoprim resistance isolates that were passed through the mouse and 20 isolates obtained following growth in liquid culture. We identified 39 unique mutations in *thyA* (data not shown) suggesting that our results were not influenced by a mutation acquired early on in the procedure. We did not observe a bias in the types of mutation from the two conditions. These results suggest that passage through the mouse results in an increase in mutation rate for *V. cholerae* suggestive of an increase in DNA damage and the need for repair mechanisms.

### Defense against RNS is required for *V. cholerae* to colonize the infant mouse

We have identified two DNA repair mechanisms required by *V. cholerae* to efficiently colonize the infant mouse, and have shown that *V. cholerae* passaged through a mouse has an increased mutation frequency. Thus, we sought to identify potential causes of DNA damage for *V. cholerae* while in the mouse to understand the requirement for BER and MMR in the mouse. A major source of DNA damage for intracellular pathogens is from host produced ROS and RNS. While *V. cholerae* is a non-invasive pathogen we considered that it still may experience ROS and RNS at some point during infection. We used a genetic approach to determine if ROS/RNS affected *V. cholerae* colonization and if so what type(s) of ROS/RNS were most important during this encounter.

Bacteria have several enzymes to detoxify ROS/RNS. Each enzyme detoxifies a specific type of ROS or RNS [for review see [18], [19]. For example catalases/peroxidases decompose H_2_O_2_, superoxide dismutases remove superoxide and ferrisiderophore reductases remove nitric oxide. Bacteria can contain multiple proteins capable of dealing with one type of stress. *V. cholerae* possesses two catalases/peroxidases (KatB/PerA) and one alkyl hydroperoxide reductase (AhpC), three superoxide dismutases (SodA/B/C) but only one ferrisiderophore reductase (HmpA). We tested mutants defective for each of these different types of defense enzymes to identify the type(s) of radicals that may be damaging *V. cholerae* in the mouse (Table 2).

**Table 2:** Ability of *V. cholerae* mutants defective in ROS or RNS detoxification to colonize the infant mouse intestine in competition with the parental strain (WT).

Gene	Function	aCompetitive Index: Tn Mutant/WT	aCompetitive Index: Deletion Mutant/WT
*ahpC*	Peroxidase	1.41±0.02	
*katB*	Catalase	1.14±0.07	
*perA*	peroxidase	1.42±0.17	
*sodA*	Superoxide dismutase	1.15±0.10	
*sodB*	Superoxide dismutase	N/A	
*sodC*	Superoxide dismutase	1.25±0.06	
*hmpA*	Ferrisiderophore reductase	0.13±0.02^***^	0.16±0.01^***^
*prxA* (VC2637)	Peroxiredoxin putative	0.14±0.02^***^	0.09±0.01^***^
*prxA/hmpA*	Double mutant		0.07±0.01^**^
*hmpA/mutS*	Double mutant		0.13±0.01^***^

a The competitive index is the ratio of mutant to parental (WT) cfu in the small intestine post infection divided by the input ratio of mutant to parental (WT) cfu. The mean and standard error of 5–10 mouse experiments is shown for each mutant. Statistical significance of the competitive colonization defect of each mutant strain relative to the null hypothesis was determined as described in the materials and methods (*** p<0.001, ** p<0.01).

**Figure 3:**
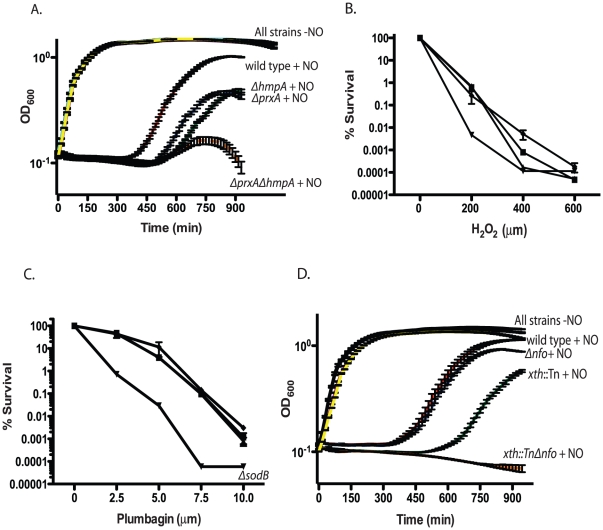
Growth of RNS/ROS mutants under stress. A. Nitric oxide stress. Exponentially growing cultures of wild type *V. cholerae*, the Δ*hmpA* mutant, Δ*prxA* mutant or Δ*prxA* Δ*hmpA* double mutant were grown with or without 1 mM spermine NONOate as a nitric oxide donor. The recovery and growth of each strain was monitored over time. The averages of 3 experiments are shown for each strain. Curves for wild type, Δ*hmpA*, Δ*prxA* and Δ*prxA* Δ*hmpA* double mutant treated with spermine NONOate are labeled as “+NO” for clarity. All strains grown in LB without spermine NONOate cluster together and are shown on the plot with the label “All strains−NO”. wild type+NO (red), Δ*hmpA*+NO (green), Δ*prxA*+NO (blue), Δ*prxA* Δ*hmpA*+NO (orange), wild type−NO (black), Δ*hmpA*−NO (purple), Δ*prxA*−NO (cyan) and Δ*prxA* Δ*hmpA*−NO (yellow). The growth of the Δ*prxA*, Δ*hmpA* and Δ*hmpA* Δ*prxA* mutant is significantly delayed by NO compared to wild type by 435 min (p<0.05). B. H_2_O_2_ sensitivity. Wild type (▪) and *katB*::Tn (▴), *perA*::Tn (▾) and *ahpC*::Tn (♦) mutants were plated on increasing concentrations of hydrogen peroxide. Cfu were determined after overnight growth. The average of 3 experiments is shown. C. Superoxide sensitivity. Wild type (▪) and *sodA*::Tn (▴), Δ*sodB* (▾) and *sodC*::Tn (♦) mutants were plated on agar containing increasing concentration of the superoxide generating compound plumbagin. Cfu were determined after overnight growth. The average of 3 experiments is shown. D. Exponentially growing cultures of wild type, the *xth*::Tn mutant, Δ*nfo* mutant or *xth*::Tn Δ*nfo* double mutant were grown with or without 1 mM spermine NONOate as a nitric oxide donor. The recovery and growth of each strain was monitored over time. The averages of 3 experiments are shown for each strain. Curves for wild type, *xth*::Tn, Δ*nfo* and *xth*::Tn Δ*nfo* treated with spermine NONOate are labeled as “+NO” for clarity. All strains grown in LB without spermine NONOate cluster together and are shown on the plot with the label “All strains−NO”. wild type+NO (red), *xth*::Tn+NO (green), Δ*nfo*+NO (blue), *xth*::Tn Δ*nfo*+NO (orange), wild type−NO (black), *xth*::Tn−NO (purple), Δ*nfo*−NO (cyan) and *xth*::Tn Δ*nfo*−NO (yellow). At 750 min the growth delay of the *xth*::Tn mutant compared to the wild type in NO is significant (p<0.001) while the growth delay of the Δ*nfo* mutant is not.

RNS, including nitric oxide, have been shown to be powerful antimicrobial agents. The most well studied RNS defense enzyme in bacteria is Hmp, a ferrisiderophore reductase that destroys nitric oxide [47]. *V. cholerae* carries an *hmp* homolog, *hmpA*. Both an *hmpA*::Tn mutant and a Δ*hmpA* deletion mutant showed a defect in colonizing the infant mouse intestine (Table 2). Deletion of *hmpA* delayed *V. cholerae* growth in the presence of a nitric oxide donor but not in the absence (Figure 3A) consistent with previous observations in other bacteria [20], [48]. This suggests that *V.cholerae* may encounter deleterious RNS during passage in the mouse. The growth defect of the Δ*hmpA* mutant in the presence of a nitric oxide donor could be complemented by ectopic expression of *hmp* from the arabinose inducible plasmid pBAD18 (Figure S1D). In fact, expression of *hmp* from pBAD18 allowed the Δ*hmpA* mutant to recover growth more rapidly than the parental strain in the presence of a nitric oxide donor.

After testing all previously predicted antioxidant enzymes we began to mine the *V. cholerae* genome for additional putative antioxidant enzymes. We began by searching for putative proteins that belonged to large antioxidant families. Enzymes, such as AhpC, belong to the Peroxiredoxin (PRX) family. Searching for peroxiredoxin family proteins yielded a putative defense enzyme we have called PrxA (VC2637). PrxA, classified is a peroxiredoxin-5 family protein, is found in several pathogenic bacteria and is a distant homolog of a macrophage peroxynitrite detoxification protein [49].

Deletion of *prxA* did not effect *V. cholerae* growth in LB alone but significantly delayed *V. cholerae* growth in the presence of a nitric oxide donor (Figure 3A). Furthermore, both the *prxA*::Tn mutant and the Δ*prxA* allele we constructed caused a decrease in the ability of *V. cholerae* to colonize the infant mouse in competition assays (Table 2). The growth defect of the Δ*prxA* mutant in the presence of a nitric oxide donor could be complemented by ectopic expression of *prxA* from the arabinose inducible plasmid pBAD18 (Figure S1E). The discovery of a new gene required for both defense against RNS and efficient colonization of the infant mouse further supports our findings that *V. cholerae* may be exposed to RNS during passage though the mouse.

We tested the sensitivity of a Δ*prxA ΔhmpA* double mutant and found that the growth of the double mutant in the presence of a nitric oxide donor was even more delayed than either the Δ*prxA* or Δ*hmpA* single mutant alone (Figure 3A). We also tested the colonization efficiency of a Δ*prxA ΔhmpA* (Table 2) and found that it was not significantly less than the Δ*prxA* mutant alone (p>0.05). Thus *hmpA* and *prxA* are both important for colonization but the effects were not additive.

We asked if defects in ROS defense also affect *V. cholerae* colonization. Disruption of *ahpC*, *katB*, *perA*, *sodA* or *sodC* did not affect the ability of *V. cholerae* to colonize the infant mouse and these deficiencies did not affect the ability of *V. cholerae* to colonize the infant mouse in competition experiments (Table 2). We did not test the *sodB*::Tn mutant since both it and a Δ*sodB* deletion strain we constructed had a decreased plating efficiency and grew very poorly compared to the parental strain (Figure S1F). Thus, while SodB appears to be important for growth of *V. cholerae* under laboratory conditions we did not pursue the *sodB* mutant in mouse experiments.

Interestingly, of *ahpC*, *katB* and *perA* only disruption of *perA* sensitized *V. cholerae* to H_2_O_2_ *in vitro* (Figure 3B). Furthermore, of strains disrupted individually for *sodA*, *sodB* and *sodC* only disruption of *sodB* sensitized *V. cholerae* to the superoxide generating compound plumbagin (Figure 3C). We also tested the Δ*prxA* mutant but found that it did not show increased sensitivity to either H_2_O_2_ or plumbagin (Figure S2A and data not shown). It is possible that some of these known ROS defense enzymes overlap in function masking the effects of a deficiency in any one gene *in vitro* or in mouse studies. For other bacterial pathogens and symbionts deletion of several or all catalases and superoxide dismutases has been required before a strong effect on virulence or symbiosis was observed [50], [51], [52]. Currently, the results from our analysis suggest RNS may pose a significant barrier to *V. cholerae* in colonizing the infant mouse. ROS may also play a role, however their effect is not immediately evident in our analysis.

XthA appears to be more important than Nfo in protecting *V. cholerae* against environmental stress *in vitro* (Figure 1B), yet Nfo appears to be more important for colonization of the small intestine (Table 1). The requirement for Δ*hmpA* and Δ*prxA* for efficient intestinal colonization lead us to ask if Nfo was required for defense against nitric oxide. We monitored the growth of the *xth*::Tn, Δ*nfo* and *xth*::Tn Δ*nfo* mutants in the presence of a nitric oxide donor. We found that, at least *in vitro*, *xth*:Tn was more important than Nfo for protection against nitric oxide (Figure 3D). We also found that the double mutant was again more sensitive to the stress than either single mutant alone (Figure 3D).

### Colonization defective mutants have increased sensitivity to acidified nitrite

Our results led us to ask if our DNA repair and RNS defense defective *V. cholerae* mutants were sensitive to any host defenses. The intestine has several innate defenses [37]. We tested many of these defenses including lysozyme, phospholipase, antimicrobial peptides, complement, bile, changes in osmolarity and pH, however, we did not observe any difference in sensitivity between the parental and the mutant strains (data not shown). RNS have been shown to be generated by macrophages to kill phagocytised bacteria [reviewed in [53]]. The RNS from macrophages is generated by an inducible nitric oxide synthase (iNOS). Inhibition of iNOS activity has been shown to rescue the virulence defects in *hmp* mutant strains of *Salmonella enterica* serovar *typhimurium* [20]. However, our *hmpA*::Tn *V. cholerae* mutant showed no difference in it ability to colonize the intestine of a wild type or isogenic iNOS^−/−^infant mouse (Table S1).

Thus, our results suggest that the colonization defect of the DNA repair and RNS defense mutants may occur before *V. cholerae* is exposed to the host defenses found in the small intestine. In the stomach *V. cholerae* is exposed to low pH in combination with µM amounts of nitrite from ingested food and the salivary nitrite cycle [25], [26], [27]. Acidified nitrite produces a variety of toxic RNS. We quantified the amount of nitrite in the infant mouse stomach using the Griess reaction and found that it was 20.0±0.7 µM, which is similar to that of humans [26]. The pH range of human gastric juice is reported as 1–3 [23], [24]. We determined the pH of the infant mouse stomach to be 4.5±0.1 using a fluorescent pH sensitive dye. This measurement is conservative and the pH of the infant mouse gastric juice may be even less (see Materials and Methods). Thus, the infant mouse stomach is sufficiently acidic to promote the formation of acidified nitrite.

**Figure 4:**
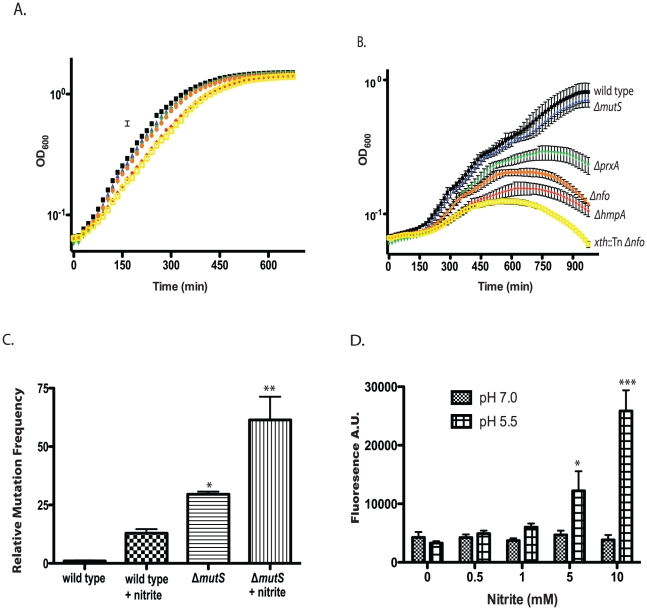
Effect of nitrite on growth of wild type and mutant *V. cholerae* strains. A/B. Exponentially growing cultures of wild type and Δ*mutS*, Δ*nfo*, Δ*prxA*, Δ*hmpA* and Δ*nfo xth*::Tn mutants were grown in LB buffered at pH 5.5 in the absence (A) or presence (B) of 400 µM sodium nitirite. The average of three experiments is shown for each strain. Wild type (black squares), Δ*mutS* (blue triangles), Δ*nfo* (orange circles), Δ*prxA* (green inverted triangle), Δ*hmpA* (red diamond) and Δ*nfo xth*::Tn (yellow open square). C. Mutation frequency as measured by rifampicin resistant colony formation frequency from wild type and Δ*mutS* mutant cultures grown at pH 5.5 in the presence or absence of 600 µM sodium nitrite. The average mutation frequency of the wild type grown in the absence sodium nitrite was normalized to 1 (* p<0.05, ** p<0.01 compared to wild type) D. Intracellular RNS production following nitrite treatment. Wild type cultures were grown at pH 7.0 or 5.5 plus 0, 0.5, 1.0, 5.0, or 10.0 mM sodium nitrite. After washing cells were exposed to the radical binding dye H_2_DCFDA. After removal of media, cells were lysed and H_2_DCFDA fluorescence was measured. The average of at least 3 independent experiments is shown with error bars representing the SEM (* p<0.05, ** p<0.01).

At pH 3 in rich medium we found that *V. cholerae* had a greater than 99.9% decrease in survival in less than 1 minute (data not shown) agreeing with similar work examining *V. cholerae* acid tolerance [54]. We did not find a difference in survival between the parental and mutant strains at low pH (1–4) levels (data not shown). We gradually increased pH to identify the lowest level at which *V. cholerae* could grow. At pH 5.5 *V. cholerae* and the DNA repair and RNS defense mutants grew with identical kinetics (Figure 4A). We titrated nitrite into the growth medium and found that nearly all the mutant strains showed a growth defect compared to the wild type at pH 5.5 in the presence of 400 µM nitrite (Figure 4B). No differences in growth between wild type and mutant strains were observed at pH 7.0 with or without 400 µM nitrite (Figure S3A, B). Not only did low pH and nitrite slow the growth of our mutants but the Δ*hmpA*, Δ*prxA*, Δ*nfo* and *xth*::Tn Δ*nfo* mutants began to show a decrease in optical density after longer exposure (Figure 4B) suggesting the cells were lysing.

Only the growth of the Δ*mutS* strain was unaffected at 400 µM. We considered that while MutS may not be required for survival of acidified nitrite during this time course it may be required to prevent acidified nitrite induced mutations in *V. cholerae* that are detrimental for colonization. We grew *V. cholerae* in LB at pH 5.5 over night in the presence or absence of 600 µM nitrite and then plated for rifampicin resistant colonies. We found that *V. cholerae* grown in the presence of nitrite had a greater than 10-fold increase in mutation frequency compared to the media only control (Figure 4C). Loss of MutS then increased the mutation frequency of *V. cholerae* in nitrite at pH 5.5 ∼ an additional 5-fold (Figure 4C). Thus, MutS may be important to prevent acidified nitrite induced mutations that could impair the ability of *V. cholerae* to colonize the infant mouse. To further test this possibility we created a Δ*hmpA* Δ*mutS* double mutant and tested its colonization proficiency (Table 2). Interestingly, the colonization defect of the Δ*hmpA ΔmutS* double mutant was not significantly different than either of the single mutants alone (p>0.05). This result may suggest that HmpA and MutS may share a similar defense pathway in the infant mouse.

Additionally, *E. coli* MutS can recognize an O6-methyl-dG:dC base pair, a mutation which can occur by alkylation of G bases [55]. Therefore it is possible that MutS may also be important for protection again some type of alkylation that occurs in the mouse stomach.

### Low pH and nitrite induce radical formation in *V. cholerae*

If acidified nitrite produces RNS that damaged *V. cholerae* DNA, we reasoned that we should be able to detect increased intracellular radical formation in *V. cholerae* following nitrite treatment. We grew *V. cholerae* at pH 5.5 with increasing amounts of nitrite and assayed for radical formation using 2′,7′-dichlorodihydrofluorescein diacetate (H_2_DCFDA). H_2_DCFDA is a cell permeable dye that fluoresces after reacting with RNS and ROS species. H_2_DCFDA reacts with several ROS and RNS including hydrogen peroxide, nitric oxide and peroxynitrite [56], [57], [58]. We found that H_2_DCFDA fluorescence correlated with increasing nitrite concentration at pH 5.5 indicating an increase in intracellular radicals (Figure 4D). For comparison, we found that H_2_DCFDA fluorescence did not increase over the concentrations of nitrate examined at pH 7.0. These results further support the requirement for low pH to induce radical formation from nitrite sources (Figure 4D).

### A colonization defect is observed for the DNA repair and RNS defense mutants early after inoculation

Our results suggested that *V. cholerae* may experience DNA damage as it passes through the stomach. If so, we were curious if a colonization defect could be observed at an early time point after inoculation. We repeated the competitive colonization assays testing the Δ*mutS*, Δ*nfo*, Δ*prxA* and Δ*hmpA* mutants. We found that at 3 hours post inoculation each mutant strain already showed a 50–60% colonization defect when co-inoculated with the wild type (Table S2) though this defect was not as great as the 5–20% defects reported in Table 1. This suggests that a defect of the mutant strains is detrimental for colonization early after inoculation. Angelichio *et al.* [59] reported that *V. cholerae* populations in the small intestine do not show a significant increase in number until between 10–24 h post inoculations. These results suggest that the effects of the DNA damage are ∼50% detrimental at the earliest stages of infection and become more apparent as the bacteria replicate to high numbers in the intestine. Such a conclusion is consistent with the concept of damage occurring primarily in the stomach.

### The colonization defect of the DNA repair and RNS defense mutants is rescued by neutralizing stomach acid

Our results support a relationship between acidified nitrite sensitivity and colonization defects in our RNS and DNA repair. We reasoned that if acidified nitrite in the stomach was responsible for the colonization defects of our mutants then neutralizing stomach acid in the mouse should relieve, at least in part, the observed colonization defects. We used sodium bicarbonate to neutralize the mouse stomach acid. When we inoculated infant mice with our DNA repair and RNS defense defective mutants in the presence of sodium bicarbonate all four mutant strains (Δ*nfo*, Δ*mutS*, Δ*prxA* and Δ*hmpA*) showed significant improvement in their ability to colonize the intestine in competition with the parental strain (Table 3). In fact the colonization defect of the Δ*mutS* and Δ*nfo* mutant was completely rescued. The colonization defect of the Δ*prxA* was restored to near wild type levels. The colonization defect of the Δ*hmpA* mutant was partially rescued although this difference is not statistically significant for the number of replicates tested (p>0.05).

**Table 3:** Effect of NaHCO_3_ on mutant *V. cholerae* ability to colonize the infant mouse intestine in competition with the parental strain (wild type).

Gene	aCompletive Index: Deletion Mutant/WT	NaHCO_3_ Treatment^a^ Competitive Index: Deletion Mutant/WT
*nfo*	0.23±0.01	1.10±0.03^***^
*mutS*	0.16±0.07	0.92±0.05^*^
*prxA* (VC2637)	0.09±0.01	0.74±0.06^**^
*hmpA*	0.16±0.01	0.45±0.01

a The competitive index is the ratio of mutant to parental (WT) cfu in the small intestine post infection divided by the input ratio of mutant to parental (WT) cfu. The average and SEM of 4–7 mouse experiments is shown for each mutant. The NaHCO_3_ treatments that causes significant rescue of the mutant colonization defect are indicated (* p<0.05, ** p<0.01, *** p<0.001). NaHCO_3_ treatment did not cause significant rescue of the Δ*hmpA* mutant (p>0.05).

The nitrite concentration of the infant mouse stomach after sodium bicarbonate treatment remained nearly unchanged at 20.3±0.8 µM.

## Discussion

We have shown that *V. cholerae* must defend against DNA damage to efficiently colonize the infant mouse intestine and that such damage likely occurs early during infection as *V. cholerae* enters the stomach. We have demonstrated that *V. cholerae* specifically requires BER and MMR pathways to efficiently colonize the infant mouse intestine. Furthermore, we have identified one previously known and one novel RNS defense protein that facilitates intestinal colonization of the infant mouse. These DNA repair and RNS defense proteins were also required for *V. cholerae* to grow or maintain genomic fidelity in the presence of acidified nitrite. Furthermore the colonization defects of each mutant could be partially or fully complemented by neutralizing stomach acid suggesting that RNS defense and DNA repair share a common defensive role in the mouse.

*V. cholerae* has been shown to be very sensitive to low pH [54]. For this reason, human volunteers have their stomach contents neutralized to promote experimental *V. cholerae* infection as is done with live attenuated vaccine studies [60]. In the recently developed infant rabbit model for cholera, stomach acid is also neutralized and cimetidine is administered to prevent re-acidification in order for *V. cholerae* to colonize the infant rabbit intestine [61]. However, our DNA repair and RNS defense mutants did not show increased sensitivity to low pH compared to the parent strain. Agreeing with our observations a large screen used to identify genes necessary for colonization and tolerating low pH in *V. cholerae* did not identify any of the genes we reported here for influencing colonization [62]. While low pH of the stomach alone is undoubtedly detrimental towards *V. cholerae* our results suggest that neutralizing stomach acid may also be important to prevent RNS formation by acidified nitrite. We propose that the defect in colonization of the DNA repair and RNS defense mutants is due to RNS formation.

Acidified nitrite is present in the stomach where gastric juice interacts from nitrite sources from the diet or salivary nitrite pathway. The chemistry of acidified nitrite is known to produce several potentially deadly radicals (1).
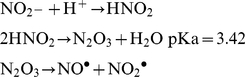
(1)Both NO^•^ and NO_2_^•^ can directly attack cellular macromolecules, but they can also interact with other radicals to form further species such as peroxynitrite and hydroxyl acids. From equation (1) we can see that a key factor in this process is the pH of the solution. In the human stomach, normal nitrite concentrations range from 10–50 µM [24] but at a pH of 1–3 acidification and radical production can happen rapidly. The low pKa of HNO_2_ may explain in part why we required much higher concentrations of nitrite to observe detrimental effects on the mutants we studied. pH 5.5 was the lowest pH level we could successfully grow *V. cholerae*, a value well above the pKa of HNO_2_. Thus at pH 5.5 the acidification of nitrite would occur more slowly and higher concentrations of nitrite would be necessary for mass action to drive the acidification and radicalization of nitrite. Agreeing with this we did not observe any effect of nitrite on the growth of our mutants or *V. cholerae* at pH 7.0. While acidified nitrite has been shown to effectively kill several bacterial pathogens [28], [29], [30], [31], the mechanism of its action and bacterial defenses to protect against it have remained unknown. We have now shown that MutS, Nfo, HmpA and PrxA are required for protection of acidified nitrite in *V. cholerae*.

While our results support a role for acidified nitrite in the stomach acting as a major DNA damaging agent, we have not identified the exact location in the gastrointestinal track where the damage occurs. The most apparent location is the stomach where ingested *V. cholerae* mixes with gastric juice. However it is possible that DNA damage induced by acidified nitrite radicals occurs, or continues to occur, in the upper intestinal tract. As gastric juice exits the stomach it is neutralized by bile salt, etc. and can reach a pH close to 8 [24]. Since the stability of at least some RNS, such as peroxynitrite, increases with increasing pH [63] the upper intestinal tract may provide a more favorable environment for RNS to reach *V. cholerae* and induce DNA damage.

Bicarbonate has been shown to induce *V. cholerae* virulence genes in a ToxT dependent fashion [64]. Abuaita and Withey show that significant upregulation of both cholera toxin and *tcpA* gene expression are observed 3–4 h after addition of bicarbonate [64]. While our inoculation of *V. cholerae* occurs on a much shorter time scale (∼5–15 min after exposure of bicarbonate) and the majority of *V. cholerae* has passed into the small intestine before 3 h, it is possible that some bicarbonate induced gene regulation may also aid in the bicarbonate rescue of the colonization defect of our DNA repair mutants.

While the debate over the benefits and detriments of increased mutation frequency for pathogenesis continues, we have shown that increased mutation frequency is detrimental to *V. cholerae* pathogenesis, at least for the short-term colonization of the infant mouse intestine. However, we cannot exclude the possibility that increased mutation frequency affects long-term survival of *V. cholerae* in the host. After the initial decrease in competitiveness it is possible that increased mutation frequency in *V. cholerae* could make it more competitive in later stages of colonization or during release into the environment. It would be interesting to test multiple clinical isolates for a mutator phenotype to address this question.

The Xth and Nfo homologs of *V. cholerae* have strong sequence similarity to their *E. coli* counterparts. We have shown that *V. cholerae* and *E. coli* deletion mutants of *xth* and *nfo* also share a similar pattern of sensitivity to hydrogen peroxide. Nfo and Xth have been most extensively studied in *E. coli*. In *E. coli* Xth is responsible for greater than ∼90% of all AP endonuclease activity in the cell [39], [40]. In *E. coli*, an *xth* mutant is very sensitive to a variety of DNA damaging agents whereas *nfo* mutants generally show milder effects [41]. Interestingly, we have shown that in *V. cholerae* Nfo is more important for colonization of the infant mouse than Xth. Our *nfo xth* double mutant suggests that Xth may play a role in colonization when Nfo is absent. However, for whatever damage is occurring, Nfo appears to play a more important role in the mouse.

It is possible that Nfo and Xth are also used differentially for repair of specific types of lesions. Preferences for specific types of damaged bases between Nfo and Xth from *E. coli* have been previously reported [65]. If RNS are responsible for DNA damage in the mouse we suggest that Nfo may have enhanced ability to aid in the repair of nitrosylative base damage. Additionally, there may be differential expression of *xth* and *nfo* or their preferential glycosylase partners in the host.

In our efforts to identify ROS and RNS defense enzymes required for intestinal colonization, we identified a new protein we have called PrxA that was required for RNS defense. Until now Hmp has been the only bacterial protein identified to detoxify RNS, specifically nitric oxide. We have shown that like HmpA, PrxA protects *V. cholerae* against the nitric oxide donor spermine NONOate. Similarly, HmpA and PrxA both protect *V. cholerae* against acidified nitrite. This agrees with previous work showing that Hmp protects *Salmonella* against nitric oxide and acidified nitrite [34]. While the species(s) produced by acidified nitrite that HmpA and PrxA defend against is not clear, we presume that it is a RNS. PrxA homologs are not as prevalent in bacteria as HmpA homologs, but they are found in several pathogens including *Yersina pestis*, *Haemphilus influenza* and *Neisseria gonorrhoeae*. It will be interesting to determine if PrxA homologs share a similar RNS defense role in other bacteria.

Our observation that HmpA and PrxA are required for colonization lead us to suggest that *V. cholerae* encounters RNS stress during infection. When studying ROS defense genes we found that deletion of SodB was detrimental for normal *V. cholerae* growth. This indicated that normal growth of *V. cholerae* must generate a significant amount of superoxide managed by SodB. The growth defect of the Δ*sodB* mutant prevented us from analyzing it by competition in the mouse model. While we did not identify any other single ROS defense enzyme that affected intestinal colonization it is possible that construction of various double mutants may show that *V. cholerae* must also deal with ROS during disease progression. In bacterial pathogens where SODs have been shown to be necessary for virulence, it is generally the periplasmic SOD that is required as this SOD encounters superoxide entering the cells from the environment [16], [66]. However, the *V. cholerae* periplasmic SOD, SodC, was not required for intestinal colonization suggesting *V. cholerae* does not experience superoxide stress from the host.

## Materials and Methods

### Ethics statement

The animal experiments were performed with protocols approved by Harvard Medical School Office for Research Protection Standing Committee on Animals. The Harvard Medical School animal management program is accredited by the Association for the Assessment and Accreditation of Laboratory Animal Care, International (AAALAC), and meets National Institutes of Health standards as set forth in the Guide for the Care and Use of Laboratory Animals (DHHS Publication No. (NIH) 85-23 Revised 1996). The institution also accepts as mandatory the PHS Policy on Humane Care and Use of Laboratory Animals by Awardee Institutions and NIH Principles for the Utilization and Care of Vertebrate Animals Used in Testing, Research, and Training. There is on file with the Office of Laboratory Animal Welfare (OLAW) an approved Assurance of Compliance (A3431-01).

### Bacterial strains

Strains and plasmids are listed in Supporting Table S3. *V. cholerae* El Tor biotype strain C6706 and a spontaneous *lacZ*− derivative of C6706, were used as parental (wild type - WT) strains. *E. coli* DH5α λpir and Sm10 λpir were used for cloning and conjugation, respectively. Antibiotic concentrations used were streptomycin (Sm: 100 µg/ml or 500 µg/ml), kanamycin (Kan: 50 µg/ml), carbenicillin (Carb: 75 µg/ml) and chloramphenicol (Cm: 2.5 µg/ml for C6706 and 10 µg/ml for *E. coli* DH5α λpir). LB was used for all growth conditions [10 g/liter of tryptone (Bacto), 5 g/liter of yeast extract (Bacto), and 5 g/liter of NaCl] and was supplemented with 16 g/liter of agar (Bacto) for growth on plates. Arabinose was used at 0.1% for complementation assays. All ID numbers/ Accession numbers/for genes highlighted in this study are shown in Table S5.

### DNA manipulations

The genomic sequence of C6706 has not been completed. We used the sequence of the close relative, N16961, for clone construction. For in-frame gene deletions of *nfo*, *mutS*, *hmpA* and *prxA*, genomic DNA surrounding the respective gene was amplified by crossover PCR and cloned into pWM91 for subsequent *sacB*-mediated allelic exchange in *V. cholerae*, as described [67], [68]. For complementation constructs, the respective gene was amplified from chromosomal DNA and cloned into plasmid pBAD18 after digestion with KpnI and SalI. The respective gene was induced by adding arabinose to the growth medium. All cloning products were sequence-verified, and the nucleotide sequence of all primers used is listed in Table S4.

### Infant mouse colonization competition assays

A modified version of the protocol of Baselski and Parker [69] was performed for infection and recovery of C6706 derived strains. C6706 or C6706 *lac* ^−^ and mutant strains were grown on LB-agar plates with Sm overnight at 37°C. Wild type and mutant strains were mixed together in LB. 50 µl of this competition mixture (∼50 000 bacteria) was inoculated into a 5-day-old CD1 mouse pup (Charles River Company). Serial dilutions of the competition mixture were plated in LB+Sm_100_ and enumerated to determine the input ratio of wild type and mutant strain. After incubation at 30°C for 3 h or 18 h the mouse pups were sacrificed and small intestines were removed and homogenized in 10 ml of LB. Serial dilutions were plated in LB+Sm_100_ and enumerated to determine the output ratio of wild type and mutant strain. The competitive index for each mutant is defined as the input ratio of mutant/wild type strain divided by the output ratio of mutant/wild type strain. A minimum of four mice were assayed for each mutant strain. The *in vivo* experiments for the transposon and clean deletion strains were the accumulation of results performed on different days. For ease of communication we reported the average competitive index.

For NaHCO_3_ recue experiments, mice pups were first inoculated with 50 µl of 2.5 g/100 mL NaHCO_3_. After 3 h the pups were inoculated with 50 µl of the competition mixture in 2.5g/ 100 mL NaHCO_3_. iNOS^−/−^ (#002609) and control C57BL/6J (#000664) mice were purchased from The Jackson Laboratory.

### Mutation frequency assays

#### Rifampicin resistance assays

i) For Δ*mutS* mutation frequency and complementation assays cultures were grown to saturation at 37°C in LB Sm_500_ or Sm_100_. 500 µl of culture was plated on LB agar+50 µg/mL rifampicin. After overnight growth at 37°C rifampicin resistant colonies were scored. ii) For mouse passaged assays, 5 day old mouse pups were inoculated with 50, 000 cells of wild type *V. cholerae*. After incubation at 30°C for 18 h the mouse pups were sacrificed and their small intestines removed and homogenized in 10 ml of LB+Sm_500_. The 10 ml of homogenized intestine was passed through cheese cloth and a 3.1µm filter. This filtering retained >90% of *V. cholerae* and removed the majority of eukaryotic materials as determined by western blot against mouse actin (data not shown). We recovered ∼250 000–500 000 *V. cholerae* cfu per small intestine. The filtrate was grown to saturation. For the control experiment 50 000 wild type *V. cholerae* were inoculated into 10 mL of LB+Sm_500_ and grown to saturation. We then plated an equal number of cfu from both mouse passaged and control cultures on LB agar+50 µg/mL rifampicin and scored resistant colonies. Control mouse samples in which no *V. cholerae* had been inoculated did not grow in LB+Sm_500_. Primers used for sequencing *rpoB* are shown in Supporting Table S3.

#### Trimethoprim resistance assays

A modified version of the Belfort and Pedersen-Lane protocol [44] was used for identified trimethoprim resistant colonies. For mouse passaged trimethoprin assays, 5 day old mouse pups were inoculated with 50 000 cells of wild type *V. cholerae*. After incubation at 30°C for 18 h the mouse pups was sacrificed and their small intestines removed and homogenized in 10 ml of LB Sm_500_+50 µg/mL thymine. The 10 ml of homogenized intestine was passaged through cheese cloth and a 3.1µm filter. This filtering retained >90% of *V. cholerae* and removed the majority of eukaryotic materials as determined by western blot against mouse actin (data not shown). We recovered ∼250 000–500 000 wild type *V. cholerae* cfu per small intestine. The filtrate was grown to saturation. For the control experiment 50 000 wild type *V. cholerae* were inoculated into 10 mL of LB Sm_500_+50 µg/mL thymine and grown to saturation. We then plated an equal number of cfu from both mouse passaged and control cultures on M9 agar+0.1% CAS, 0.2% glucose, 50 µg/mL thymine and 20 µg/mL trimethoprim. After overnight growth at 37°C trimethoprim resistant colonies were scored. Control mouse samples in which no *V. cholerae* had been inoculated did not grow in LB+Sm_500_. The nucleotide sequences of the primers used for sequencing *thyA* are shown in Supporting Table S4.

To calculate the relative mutation frequency we plated equal numbers of cfu for both mouse and passaged and control samples. We calculated the average and standard error for the mutation rate for the control samples. Next we normalized the individual mutation frequencies from our 5 mice passaged samples and 5 control samples to the average control sample mutation frequency. This normalized the average control sample mutation frequency to 1 and showed the relative mutation frequency increase in mouse passaged samples.

### Stomach pH and nitrite concentration determination

We have developed a fluorescence based assay to determine the pH of the infant mouse stomach. We first determined a standard curve using the fluorescent pH indicator Yellow/Blue DND-160 (Invitrogen) over a range from pH 3–8. We then extracted the gastric juice from 5 individual mice, diluted the sample 1∶2 with ddH_2_O (pH 7), added Yellow/Blue DND-160 and determined the fluorescence of the solution. Comparing these fluorescent values to our standard curve we determined the pH of the infant mouse stomach to be 4.5±0.1. We also note that this is a conservative measurement. In order to obtain enough liquid we diluted the gastric sample ∼1∶2 with ddH_2_0 that was at ∼pH 7. Thus while water is not a buffer, the dilution of the gastric juice likely raised the final pH of our measurements.

Nitrite concentration was determined using the Griess Reagent System (Promega TB229). The concentration shown is the average of 10 mice treated with or without sodium bicarbonate.

### Hydrogen peroxide sensitivity assays

Strains were grown to exponential phase in LB with Cm when required. Strains were serial diluted and spotted on LB plates containing increasing concentrations of hydrogen peroxide and incubated at 37°C overnight. For complementation Cm and arabinose were added while strains were growing in liquid, as well as in the LB agar plates.

### Nitric oxide sensitivity growth curves

Strains were grown to exponential phase in LB. Strains were then diluted to OD_600_ 0.01 in LB±1.0 mM spermine NONOate and grown at 37°C in a 96 well plate with aeration (SpectraMax Plus 384, Molecular Devices). OD_600_ readings were taken every 15 min.

### Growth in acidified nitrite

Overnight cultures were diluted into LB and grown to log phase at 37°C with aeration. Cultures were diluted to OD_600_ 0.05 in 25 mM MES buffered LB of pH 7.0 or 5.5 with or without the addition of 400 µM sodium nitrite (Sigma-Aldrich). The LB media and MES were adjusted to a pH of 7.0 and 5.5 (Corning pH meter 240) with additions of HCl, and filter sterilized (0.22 µm, Corning) prior to use. The growth of strains under various treatments were determined by OD_600_ measurement using a 96 well format spectrophotometer (SpectraMax Plus 384, Molecular Devices). Environmental parameters were set to 37°C with shaking and readings were taken every 15 minutes for 16 hours. Studies were conducted in quadruplicate.

### Fluoroscein assay

Overnight cultures were diluted into 100 mL LB with Sm_100_ and grown to OD_600_∼0.8 (37°C, aeration). 10 mL of culture was dispensed into 15 mL conical and centrifugated at 5,000 RPM for 5 minutes. The supernatant discarded and cells resuspended in an equal volume of 25 mM MES buffered LB of pH 7.0 or 5.5 with or without the addition of sodium nitrite (500 µM, 1 mM, 5mM, or 10 mM). Cells were treated for 1.5 hours at 37°C with aeration then centrifugated at 5,000 RPM for 5 minutes at 4°C. The supernatant was discarded, cells resuspended in 1 mL PBS (LONZA), and transferred to a 1.5 mL eppendorf tube. The cells were centrifugated and washed an additional two times in 1× PBS before being resuspended in 1 ml of PBS with 10 µM 2′,7′-dichlorodihydrofluorescein diacetate (Molecular Probes, Invitrogen). The cells were incubated at room temperature for 30 minutes then centrifugated and washed three times to remove all free, extracellular dye. The cells were lysed in 225 µL of lysis buffer (MilliQ water with 0.1M EDTA) via sonication. Cell lysates were centrifugated at 15,000 RPM for 5 minutes, supernatant transferred to another 1.5 mL eppendorf tube and centrifugated again. Fluorescence was measured at 490 nm / 519 nm (excitation/emission) (SpectraMax Gemini XS). Fluorescence was normalized against protein concentrations, as determined by Bradford assay. Studies were conducted in triplicate.

### Statistical methods

Statistical significance was assessed for mouse colonization assays and Δ*mutS* mutation frequency assays using a one-way analysis of variance (ANOVA) using a Bonferroni post test to determine significant differences in competitive index between all pairs of *V. cholerae* mutants used in our study. Statistical significance of acidified nitrite, nitric oxide and H_2_O_2_ sensitivities was assessed using a mixed model, repeated measures two-way analysis of variance (ANOVA), generating a p value for each pair wise curves over the concentration range of H_2_O_2_ to determine the significance of our results. Statistical significance of rifampicin and trimethoprim resistant mutants from LB vs. mouse passaged samples were assessed using a paired t-test. Differences were considered significant at p<0.05. All calculations were performed using Graphpad Prisim version 5.

## Supporting Information

Figure S1 A. Growth of wild type (black square), Δ*mutS* (red triangle), Δ*nfo* (blue circle) and *xth*::Tn Δ*nfo* (green diamond) in LB at pH 7.0. Growth was measured by changes in the cultures OD_600_ readings. The averages of 3 experiments are shown for each strain. B. Complementation of the mutator phenotype of the Δ*mutS* mutant. The number of wild type+pBAD18 colonies was normalized to 1. The averages of 3 experiments are shown (*** p<0.001). C. Complementation of H_2_O_2_ sensitivity of the Δ*nfo xth*::Tn mutant by overexpression of Nfo. Wild type+pBAD18 (▪), Δ*nfo xth*::Tn mutant+pBAD18 (▴) and Δ*nfo xth*::Tn mutant+p*nfo* (▾). The averages of 3 experiments are shown. D. Complementation of Δ*hmpA* mutant nitric oxide sensitivity. Exponentially growing cultures of wild type+pBAD18 (black squares), Δ*hmpA* mutant+pBAD18 (blue circles) and Δ*hmpA* mutant+p*hmpA* (green triangles) were grown with 1 mM spermine NONOate as a nitric oxide donor. The recovery and growth of each strain was monitored over time. The averages of 3 experiments are shown for each strain. E. Complementation of Δ*prxA* mutant nitric oxide sensitivity. Exponentially growing cultures of wild type+pBAD18 (black squares), Δ*prxA* mutant+pBAD18 (blue circles) and Δ*prxA* mutant+p*prxA* (green triangles) were grown with 1 mM spermine NONOate as a nitric oxide donor. The recovery and growth of each strain was monitored over time. The averages of 3 experiments are shown for each strain. F. Growth of wild type (•) and the Δ*sodB* (▪) mutant in LB at pH 7.0. Growth was measured by changes in the cultures OD_600_ readings. The averages of 3 experiments are shown for each strain.
(0.68 MB EPS)

Figure S2 A. Hydrogen peroxide sensitivity. Wild type (•) and Δ*prxA* mutant (▪) were plated on agar containing increasing concentration of the hydrogen peroxide (H_2_O_2_). Cfu were determined after overnight growth. The average of 3 experiments is shown.
(0.43 MB EPS)

Figure S3 Exponentially growing cultures of wild type and Δ*mutS*, Δ*nfo*, Δ*prxA*, Δ*hmpA* and Δ*nfo xth*::Tn mutants were grown in LB buffered at pH 7.0 in the absence (A) or presence (B) of 400 µM sodium nitrite. Growth was measured by changes in the cultures OD_600_ readings. The average of three experiments is shown for each strain. Wild type (black squares), Δ*mutS* (blue triangles), Δ*nfo* (orange circles), Δ*prxA* (green inverted triangle), Δ*hmpA* (red diamond) and Δ*nfo xth*::Tn (yellow open square).
(0.56 MB EPS)

Table S1 Colonization of C57B and isogenic iNOS−/− infant mice by the hmp::Tn mutant.
(0.02 MB DOC)

Table S2 Ability of *V. cholerae* mutants defective in DNA repair pathways to colonize the infant mouse intestine in competition with the parental strain (WT) 3 h post inoculation.
(0.03 MB DOC)

Table S3 Bacterial strains and plasmids used in this study.
(0.06 MB DOC)

Table S4 Primers used in this study.
(0.03 MB DOC)

Table S5 ID numbers/ Accession numbers for genes used in this study.
(0.03 MB DOC)
