# Supplementary material for: DNA Damage and Reactive Nitrogen Species are Barriers to Vibrio cholerae Colonization of the Infant Mouse Intestine
Source: PLoS Pathog. 2011 Feb 17;7(2):e1001295. doi: 10.1371/journal.ppat.1001295 (PMC3040672; doi:10.1371/journal.ppat.1001295)
Supplement: Table S1 — Colonization of C57B and isogenic iNOS−/− infant mice by the hmp::Tn mutant. (0.02 MB DOC) [file ppat.1001295.s004.doc]

**Table S1. Colonization of C57B and isogenic iNOS^-/-^ infant mice by the *hmp*::Tn mutant.**

| **Mutant** | **^a^Competitive Index from C57B wild type mice** | **^a^Competitive Index from iNOS^-/-^ mice** |
| --- | --- | --- |
| *hmp*::Tn | 0.23 ± 0.03 | 0.18 ± 0.03 |

^a^ The competitive index is the ratio of the *hmp*::Tn mutant to parental (WT) cfu found in the small intestine after 18h divided by the input ratio of the *hmp*::Tn mutant to parental (WT) cfu. The average and standard error of 5 mouse experiments per condition is shown.
