# Supplementary material for: DNA Damage and Reactive Nitrogen Species are Barriers to Vibrio cholerae Colonization of the Infant Mouse Intestine
Source: PLoS Pathog. 2011 Feb 17;7(2):e1001295. doi: 10.1371/journal.ppat.1001295 (PMC3040672; doi:10.1371/journal.ppat.1001295)
Supplement: Table S2 — Ability of V. cholerae mutants defective in DNA repair pathways to colonize the infant mouse intestine in competition with the parental strain (WT) 3 h post inoculation. (0.03 MB DOC) [file ppat.1001295.s005.doc]

**Table S2. Ability of *V. cholerae* mutants defective in DNA repair pathways to colonize the infant mouse intestine** in competition with the parental strain (WT) 3 h post inoculation.

| **Gene** | **Function** | **Repair Pathway** | **^a^Competitive Index:**  **Deletion Mutant/WT** |
| --- | --- | --- | --- |
| *mutS* | Mismatch Recognition | Mismatch Repair | 0.47 ± 0.04 |
| *nfo* | Endonuclease IV | Base Excision Repair | 0.53 ± 0.06 |
| *hmpA* | Ferrisiderophore reductase | Nitric oxide defense | 0.45 ± 0.14 |
| *prxA* (VC2637) | Peroxiredoxin putative | Nitric oxide defense | 0.43 ± 0.04 |

**^a^** The competitive index is the ratio of mutant to parental (WT) cfu in the small intestine post infection divided by the input ratio of mutant to parental (WT) cfu. The average and standard error of 3-5 mouse experiments is shown for each mutant.
