# Supplementary material for: DNA Damage and Reactive Nitrogen Species are Barriers to Vibrio cholerae Colonization of the Infant Mouse Intestine
Source: PLoS Pathog. 2011 Feb 17;7(2):e1001295. doi: 10.1371/journal.ppat.1001295 (PMC3040672; doi:10.1371/journal.ppat.1001295)
Supplement: Table S3 — Bacterial strains and plasmids used in this study. (0.06 MB DOC) [file ppat.1001295.s006.doc]

**Table S3.** Bacterial strains and plasmids used in this study.

| Strain/plasmid | Relevant genotype and property | Source |
| --- | --- | --- |
| Strain  SM10λpir  C6706  C6706 *lac^-^*  EC6464  EC18835  EC20580  EC17580  EC16137  EC8686  EC24371  EC9910  EC15957  EC12940  EC17417  EC16198  BDJM1  BDJM2  BDJM3  BDJM4  BDJM5  BDJM6  BDJM7  BDJM8  BDJM9  BDJM110  BDJM11  BDJM12  BDJM13  BDJM14  BDJM15  BDJM16  BDJM17  BDJM18  Plasmid  pWM19  pBAD18-Cm  p*nfo*  p*mutS*  *phmpA*  p*prxA* | *thi thr leu tonA lacY supE recA*::RP4-2-Tc::Mu 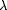*pirR6K* Km^r^  V. cholerae El Tor biotype, Sm^R^  V. cholerae El Tor biotype, Sm^R^, *lacZ^-^*  C6706 *lac^-^*, *uvrA*::Tn  C6706 *lac^-^*, *xth*::Tn  C6706 *lac^-^*, *nfo*::Tn  C6706 *lac^-^*, *mutS*::Tn  C6706 *lac^-^*, *ahpC*::Tn  C6706 *lac^-^*, *katB*::Tn  C6706 *lac^-^*, *perA*::Tn  C6706 *lac^-^*, *sodA*::Tn  C6706 *lac^-^*, *sodB*::Tn  C6706 *lac^-^*, *sodC*::Tn  C6706 *lac^-^*, *hmpA*::Tn  C6706 *lac^-^*, *prxA*::Tn (VC2637::Tn)  C6706, ∆*nfo*  C6706, ∆*mutS*  C6706 *lac^-^*, ∆*nfo* *xth*::Tn  C6706, ∆*sodB*  C6706, ∆*hmpA*  C6706, ∆*prxA* (VC2637)  C6706 pBAD18  C6706 *lac^-^*, ∆*nfo* *xth*::Tn pBAD18  C6706 *lac^-^*, ∆*nfo* *xth*::Tn p*nfo*  C6706, ∆*mutS* pBAD18  C6706, ∆*mutS* p*mutS*  C6706, ∆*hmpA* pBAD18  C6706, ∆*hmpA* p*hmp*  C6706, ∆*prxA* pBAD18  C6706, ∆*prxA* p*prxA*  C6706, ∆*nfo* ∆*mutS*  C6706, ∆*hmpA* ∆*mutS*  C6706, ∆*prxA* ∆*hmpA*  carb^R^, for creating gene deletions  arabinose inducible promoter, cat^R^  pBAD18 expressing Nfo  pBAD18 expressing MutS  pBAD18 expressing HmpA  pBAD18 expressing PrxA | [1]  [2]  Lab stock  [3]  [3]  [3]  [3]  [3]  [3]  [3]  [3]  [3]  [3]  [3]  [3]  This study  This study  This study  This study  This study  This study  This study  This study  This study  This study  This study  This study  This study  This study  This study  This study  This study  This study  This study  [4]  This study  This study  This study  This study  This study |

**Supporting References**

1. Miller VL, Mekalanos JJ (1988) A novel suicide vector and its use in construction of insertion mutations: osmoregulation of outer membrane proteins and virulence determinants in Vibrio cholerae requires toxR. J Bacteriol 170: 2575-2583.

2. Roberts A, Pearson GD, Mekalanos JJ. Cholera vaccines strains derived from a 1991 Peruvian isolate of Vibrio cholerae and other El Tor strains; 1992.

3. Cameron DE, Urbach JM, Mekalanos JJ (2008) A defined transposon mutant library and its use in identifying motility genes in Vibrio cholerae. Proc Natl Acad Sci U S A 105: 8736-8741.

4. Metcalf WW, Jiang W, Daniels LL, Kim SK, Haldimann A, et al. (1996) Conditionally replicative and conjugative plasmids carrying lacZ alpha for cloning, mutagenesis, and allele replacement in bacteria. Plasmid 35: 1-13.
