# Supplementary material for: DNA Damage and Reactive Nitrogen Species are Barriers to Vibrio cholerae Colonization of the Infant Mouse Intestine
Source: PLoS Pathog. 2011 Feb 17;7(2):e1001295. doi: 10.1371/journal.ppat.1001295 (PMC3040672; doi:10.1371/journal.ppat.1001295)
Supplement: Table S4 — Primers used in this study. (0.03 MB DOC) [file ppat.1001295.s007.doc]

**Table S4.** Primers used in this study

| Primers for in frame deletions  Nfo15´ atactcgagcgattacagagattcagtgaaagtacgcgc  Nfo25´ gatgccaaatctgtgaagtttgccgattaattctccatattacgacttcttattgttgctaaatgacttcg  Nfo13´ cgaagtcatttagcaacaataagaagtcgtaatatggagaattaatcggcaaacttcacagatttggcatc  Nfo23´ atactcgagtgggatttgaaatccagggatatcttgcgc  MutS15´ atactcgagcgcgcattatcgctcaaccgggcc  MutS25´ ccatgcttgagatatgggcaataactagagtttcatcataatcttatgtcgctgcttatc  MutS13´ gataagcagcgacataagattatgatgaaactctagttattgcccatatctcaagcatgg  MutS23´ atactcgagaccattcgtctaccgattcatgtcgtc  HmpA15´ atagggcccaataggcgatgaaaacgctcttcgttg  HmpA25´ gcatgggcgtcgcgccaacaccagttattatggggctaattcacgcaaagtctccag  HmpA13´ ctggagactttgcgtgaattagccccataataactggtgttggcgcgacgcccatgc  HmpA23´ atactcgagcttcagcgcaatccaaggtgacgg  PrxA15´ atagggccccaagcatgatcgcaaggcactgc  PrxA25´gctcccggatggaagcctagaaacttagttcctcatttgtgaaatttaattttaaatcaataggatg  PrxA13´ catcctattgatttaaaattaaatttcacaaatgaggaactaagtttctaggcttccatccgggagc  PrxA23´ atactcgagcgatgcagggattggccaagctc  SodB15´ atactcgagtggaacatttccagcatgatgtcacaaccg  SodB25´ caggcttatgacgatttattaagcagtattagcttatgccattgctcgattctccgttgagtg  SodB13´ cactcaacggagaatcgagcaatggcataagctaatactgcttaataaatcgtcataagcctg  SodB23´ ataggatcctacgtagttgatcttggcgataggcgg  Primers for complementation  Nfo5´ ataggtaccaggaggaaacgatgtatgagaagttaatcggggcacatgtatcg  Nfo3´ atagtcgacttaattctctttttgcgtcgaaaatttgcgtagcgttgc  MutS5´ atagctagcaggaggaaacgatgatgaaatcgaacgcctcaccgagcg  MutS3´ atagtcgacctagagcagctttttcaattgatagagcatatctagagc  HmpA5´ atagctagcaggaggaaacgatgctcacccaagaacacatcaatatcattaaaagcacc  HmpA3´ atagtcgacttacgccgcaagctgtgcatgtggacc  PrxA5´ ataggtaccaggaggaaacgatgaggaacacaatgtttacatctaaagaaggtc  PrxA3´ ataaagcttttattgatttaggtagacttctaagtcgtcgc  Primers for *rpoB* and *thyA* sequencing  thyA5´ gggcaaatcctctccttacctatggtg  thyA3´ agcattcttgccctaaccgcttaagcg  rpoB15´cagcgattagtcactgtcctcaccc  rpoB25´ gtcgtgtgactgcacgtcacatccgtg  rpoB35´ actgacgaagtggattacctctctgcg  rpoB45´ gcgatggcgtagaaaaagacaagcgtgcgc  rpoB13´ agatactttacctacgatgtactcaaccgg  rpoB23´ ccttcttctgtcagcttagcgttcgcc  rpoB33´ ccttccagaatttggaactcttcagtcagg  rpoB34´ tagctcgatgttgatacccagcgagcg |
| --- |
