# Supplementary material for: DNA Damage and Reactive Nitrogen Species are Barriers to Vibrio cholerae Colonization of the Infant Mouse Intestine
Source: PLoS Pathog. 2011 Feb 17;7(2):e1001295. doi: 10.1371/journal.ppat.1001295 (PMC3040672; doi:10.1371/journal.ppat.1001295)
Supplement: Table S5 — ID numbers/ Accession numbers for genes used in this study. (0.03 MB DOC) [file ppat.1001295.s008.doc]

**Table S5. ID numbers/ Accession numbers/for genes used in this study**

| **Gene** | ***V. cholerae* Gene ID Number** | **LocusLink Protein Accession Number** |
| --- | --- | --- |
| *uvrA* | VC0394 | AAF93567 |
| *xth* | VC1860 | AAF95008 |
| *nfo* | VC2360 | AAF95503 |
| *mutS* | VC0535 | AAF93703 |
| *ahpC* | VC0731 | AAF93896 |
| *katB* | VC1585 | AAF94739 |
| *perA* | VC1560 | AAF94714 |
| *sodA* | VC2694 | AAF95835 |
| *sodB* | VC2045 | AAF95193 |
| *sodC* | VC1583 | AAF94737 |
| *hmpA* | VCA0183 | AAF96096 |
| *prxA* | VC2637 | AAF95778 |
